# Maternal, Fetal, and Placental Selectins in Women With Pre-eclampsia; Association With the Renin-Angiotensin-System

**DOI:** 10.3389/fmed.2020.00270

**Published:** 2020-06-12

**Authors:** Hiten D. Mistry, Melissa V. Hott Ogalde, Fiona Broughton Pipkin, Geneviève Escher, Lesia O. Kurlak

**Affiliations:** ^1^Department of Obstetrics and Gynaecology, University of Nottingham, Nottingham, United Kingdom; ^2^Department of Nephrology and Hypertension, University of Bern, Bern, Switzerland; ^3^Department of Biomedical Research, University of Bern, Bern, Switzerland

**Keywords:** selectins, angiotensin receptors, endothelial dysfunction, pre-eclampsia, inflammation

## Abstract

Selectins [endothelial (E), platelet (P), and leucocytes (L)] are a class of cell adhesion molecules, stimulated in response to inflammation. Pre-eclampsia is characterized by inflammation, and angiotensin II is pro-inflammatory. We hypothesized that circulating maternal and fetal concentrations and placental expression of selectins would be increased in women with pre-eclampsia and would be associated with the angiotensin receptors (AT1R and AT2R). Maternal and fetal blood and placental tissue was collected at delivery from White European normotensive controls (*n* = 17) and women with pre-eclampsia (*n* = 17). Soluble (s) E-, P- and L-selectin protein concentrations were measured by ELISA and placental protein expression was examined by immunohistochemistry. Maternal sE-selectin concentrations were increased in pre-eclampsia (*P* < 0.001); conversely fetal sE- and sP-selectin levels were lower in pre-eclampsia (*P* < 0.05 for both). Staining was mainly localized to the syncytiotrophoblast for all selectins. E-selectin expression was increased, while P-selectin was decreased in placental from pre-eclampsia (*P* < 0.05 for both); no differences were observed for L-selectin expression. Both E- and L-selectin were positively correlated (*P* < 0.008; *P* < 0.02) with AT2R placental expression, whilst P-selectin was negatively associated with AT1R (*P* < 0.005), all only in the pre-eclampsia group. This novel study reports maternal, fetal and placental expression of selectins in pre-eclampsia. The increased E-selectins reflect the endothelial dysfunction, characteristic of pre-eclampsia. In contrast, the reduced P-selectins and the positive association of placental AT2Rs with both E-and L-selectin in pre-eclampsia could be a protective mechanism to limit the endothelial dysfunction.

## Introduction

Pre-eclampsia is a pregnancy-specific syndrome characterized by *de novo* hypertension and proteinuria after 20 weeks gestation (1). It affects between 2 and 8% of all pregnancies and is a major cause of both maternal and perinatal morbidity and mortality worldwide (2, 3). The consequences of pre-eclampsia are not restricted to pregnancy, with both mothers and their offspring being at increased in risk of cardiovascular, metabolic and renal disease in later life (4, 5). The etiology of pre-eclampsia remains unknown, but endothelial dysfunction due to abnormal placentation, oxidative stress, extensive leucocyte activation and inappropriate activation of the renin-angiotensin system (RAS) appear to be key contributors (6–10). The endothelium is the main regulator of vascular homeostasis. In addition to contributing to vasodilation, the intact endothelium also has anti-aggregatory, anti-inflammatory and anti-proliferative properties (11). Hypertension is associated with endothelial dysfunction, which in turn results from a dysregulation of locally-acting mediators, such as the vasodilators nitric oxide, and prostacyclin and the vasoconstrictor endothelin, and angiotensin II (Ang II)

The RAS is an important regulator of blood pressure and is one of the first hormonal systems to recognize pregnancy (10). Moreover, the RAS also plays a role in various pathological situations involving regulation of cell proliferation, inflammation, oxidative stress, and angiogenesis (12, 13). We have previously demonstrated a disrupted placental RAS in pre-eclampsia with the angiotensin II type 1 receptor (AT1R), activation of which results in vasoconstriction, being more highly expressed in placenta from women with pre-eclampsia (14).

Selectins are type 1 transmembrane proteins and are involved in leucocyte trafficking, mediating the initial tethering and rolling interactions between blood and lymphatic organs via the endothelium (15). Selectins were so named because of their ability to selectively bind carbohydrate moieties via their ligand P-selectin glycoprotein ligand-1 (PSGL-1) (15). There are three types of selectins, endothelial E-, platelets P- and leucocyte L-selectins; CD62E, CD62P and CD62L, respectively. All 3 selectins are composed of common extracellular domains and are distinguished from one another by their variable number of consensus repeats and very different cytoplasmic tails (16). E-selectin is normally absent from resting endothelium, but is transcriptionally induced in venules of multiple organs by inflammatory mediators, such as tumor necrosis factor (TNF) and IL-1 (17). P-selectin is constitutively synthesized in both platelets and endothelial cells and stored in Weibel-Palade bodies. They are rapidly translocated to the surface upon stimulation with inflammatory mediators, such as thrombin, histamine, and platelet-activating factor (17).

The shortest selectin molecule, present in leukocytes, is L-selectin, which, in the microvasculature, facilitates neutrophil rolling and migration during inflammatory responses (18). Elevated sE-selectin levels may be indicative of endothelial cell activation and damage. In addition to the conventional roles described above, the selectins play an essential role in human implantation; L-selectin and its ligands participate in the adhesion of the blastocyst to the endometrium at the maternal-fetal interface (19). Moreover, P- and E-selectins are also involved in immune recognition between maternal decidua and the embedded embryo, as well as trophoblast migration within decidual spiral arterioles (19). Reduced expression of all selectins has been reported in both decidual cells in uterine decidual stroma and in the placenta (cytotrophoblasts and syncytiotrophoblasts) of early miscarriages (6–11 weeks) compared to controls (terminations for social reasons) (20).

Concentrations of both soluble (s) E- and P-selectins have been reported to be increased in patients with chronic hypertension with the most severe endothelial dysfunction (21). Previous studies have reported elevated maternal plasma sE-selectin concentrations in women with pre-eclampsia, both at the time of delivery (22–26) and also prior to clinical diagnosis (22). However, the available data concerning both sP- and sL-selectins (22, 23, 26, 27) are conflicting.

Associations between the RAS and the selectins have previously been reported: Ang II is a potent stimulus to angiogenesis, both directly and by supplying angiogenic factors by platelet aggregation (28) and inducing the expression of P-selectin on platelets, while lessening its expression in endothelial cells (29). In addition, platelets express AT1R on their surface, and AT1R antagonists reduce P-selectin expression in platelets (30) and inhibit P-selectin-mediated platelet adhesion to the microvessels (31). Ang II also acts directly on naive T cells inducing upregulation of L-selectin via the AT1R (32). Finally, Ang II treatment significantly increased E-selectin mRNA expression in vascular endothelial cells collected from lung tissues in a mouse model of pulmonary metastasis (33).

We hypothesized that both soluble and placental expression of selectins would be increased in women with pre-eclampsia reflecting the endothelial dysfunction and inflammation characteristic of the syndrome. These selectins would also be associated with RAS receptors, contributing to the mechanistic changes associated with pre-eclampsia. This study therefore aimed to measure both soluble and placental expression of all selectins in matched maternal, fetal, and placental samples from normotensive control women and women with pre-eclampsia.

## Materials and Methods

### Subjects and Sample Collection

The study population consisted of 17 normotensive control women and 17 women with pre-eclampsia, collected over a period of 18 months (Table 1). Nottingham University Hospital Ethics Committee approved the investigations and written informed consent to participation was obtained from each woman. Cases were defined on admission with a clinical diagnosis of pre-eclampsia, defined as a systolic blood pressure of ≥ 140 mm Hg and diastolic pressure (Korotkoff V) of ≥ 90 mm Hg on 2 occasions after 20 weeks' gestation in a previously normotensive women and proteinuria > 300 mg/L, 500 mg/day or 2+ on dipstick analysis of midstream urine (MSU) if 24-h collection result was not available (1). Although samples were collected under the criteria defined originally (1), they still fit the recently updated definition (34). For subgroup analysis, the pre-eclampsia group was further split by early- (diagnosis ≤ 34 weeks) and late- (diagnosis >34 weeks) onset pre-eclampsia (35). Medical and obstetric histories, including delivery data, were obtained for each woman. The birthweight centile for each baby was computed, correcting for gestation age, sex, maternal parity, and body mass index (BMI) (36). Venous blood samples were collected before delivery and umbilical venous (fetal) blood were collected immediately after and processed as previously described (6); samples were stored in aliquots at −80^o^C until analysis. In addition, placental tissue samples were collected from a standardized location midway between cord insertion and placental border and processed for immunohistochemistry as previously described (6).

**Table 1:** Clinical and obstetric data of subject groups^*^.

**Parameter**	**NC (*n* = 17)**	**PE (*n* = 17)**
Age (yrs)	28.2 ± 7.2	31 ± 6.5
Booking body mass index (Kg/m^2^)	26.4 ± 5.5	24.8 ± 3.5
Smoking status		
Non-smoker	9 (53)	11 (65)
Smoker	8 (47)	6 (35)
Parity		
Nulliparous	11 (65)	10 (59)
Multiparous	6 (35)	7 (41)
Max. systolic blood pressure (mmHg)	114 ± 3.8	155 ± 3.8
Max. diastolic blood pressure (mmHg)	75 ± 2.1	97 ± 5.0
Proteinuria (g/L) Median [min, max]	-	1.0 [0.3, 9.4]
Gestational age at delivery (Wks)	40.0 ± 1.0	37.7 ± 1.8
Mean birthweight (g)	3439 ± 498	2993 ± 735
Corrected birthweight centile	27.9 [17.5, 66.8]	35.1 [8.6, 76.3]
Caesarean section	4 (24)	13 (76)
Early-onset PE	-	6 (35)

* *Data represented as means ± SD or median [IQR] as appropriate, except for smoking status, parity and Caesarean sections and early-onset PE, which are shown as number (percentage). NC, normotensive control; PE, pre-eclampsia; BMI, body mass index. P < 0.05 between normotensive controls and women with pre-eclampsia*.

### Soluble Selectin Assays

Soluble (s) E-, P-, and L-selectin were measured in maternal and fetal EDTA plasma using ELISAs (KA0116, KA0548, and KA0117, respectively; Abnova, Taiwan) following the manufacturers' instructions. Plasma samples were diluted 1:100, run in duplicate and blinded to outcome group. Intra- and inter-assay variations were: sE-selectin: 5.4 and 6%; sP-selectin: 5.5 and 6.1%; sL-selectin: 3.7 and 4.2%.

### Placental Selectin Protein Expression/Localization

Immunohistochemical analysis was performed as previously described (37), using antibodies to E-selectin (mouse monoclonal, BBA16; 5 μg/mL; R&D Systems), P-selectin (mouse monoclonal, BBA30; 10 μg/mL; R&D Systems) and L-selectin (rabbit polyclonal, GTX59778; 0.1 μg/mL; GeneTex Inc.). All slides were assessed by the same observer, blinded to pregnancy outcome. Quantification was performed as described previously (12), using the Positive Pixel Algorithm of Aperio ImageScope software; a visual check was also performed to establish localization of staining. The AT1R and AT2R protein expression analysis was performed as previously reported (14).

### Statistical Analysis

All tests were performed using SPSS version 24. Summary data are presented as means ± standard deviation (SD) or median and interquartile range (IQR) as appropriate. The Kolmogorov-Smirnov test indicated the experimental data were not normally-distributed. The Kruskal-Wallis test followed by Mann-Whitney *U*-test was used for multiple group analysis; the Wilcoxon paired tests were used for maternal and fetal selectin concentrations. Potential associations between continuous data were tested using Spearman's Rho correlation tests. The null hypothesis was rejected where *P* < 0.05.

## Results

### Subjects

Demographics and clinical characteristics of the participants are summarized in Table 1. By definition, blood pressures were significantly increased (*P* < 0.05) and significant proteinuria was present in the pre-eclampsia group. Birthweights were also lower in the women who suffered from pre-eclampsia. The groups were matched for maternal age, BMI, smoking status, parity, and gestational age at delivery.

### Soluble Selectin Concentrations in Maternal and Fetal Plasma

**Figure 1:**
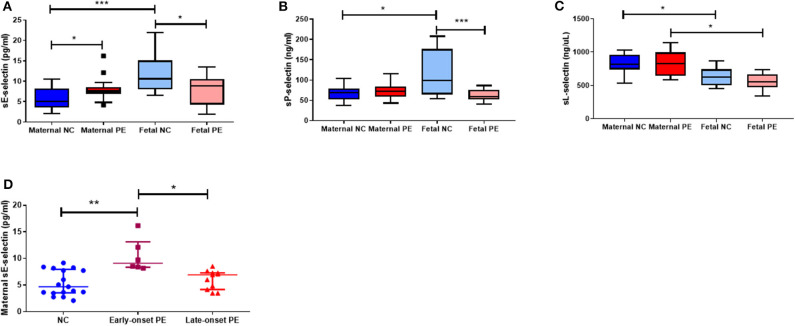
Maternal and fetal soluble (s) **(A)** E-selectin; **(B)** P-selection and **(C)** L-selectin concentrations between normotensive control (NC) and pre-eclamptic (PE) pregnancies. **(D)** Maternal sE-selectin concentrations when sub-grouped by NC, early-onset PE (diagnosis ≤ 34 weeks gestation; *n* = 6) and late-onset PE (>34 weeks gestation; *n* = 11). Data presented as median [IQR]; *P < 0.05; ***P < 0.0001.

Concentrations of all soluble selectins are summarised in Figure 1. Maternal sE-selectin was increased in pre-eclampsia compared to controls (*P* < 0.05; Figure 1A). No significant differences were observed for sP- or sL-selectins (*P* > 0.05 for both; Figures 1B,C). In contrast, in the fetus, both sE- and sP-selectin concentrations were lower in the pre-eclampsia group (*P* < 0.05 for both; Figures 1A,B), but did not differ for sL-selectin (*P* > 0.05; Figure 1C).

When comparing paired maternal-fetal samples, both sE- and sP-selectin concentrations were increased in fetal compared to maternal plasma only in the normotensive group (*P* < 0.05 for both; Figures 1A,B); fetal plasma from women who had pre-eclampsia remaining similar to their paired maternal plasma (*P* > 0.05). Conversely, fetal sL-selectin concentrations were reduced compared to their paired maternal samples in both groups (*P* < 0.05; Figure 1C).

When sub-grouped by early-/late-onset pre-eclampsia, only maternal sE-selectin concentrations were raised in plasma from early-onset (*n* = 6) pre-eclampsia (*P* = 0.003), compared to both late-onset (*n* = 11) pre-eclampsia (*P* = 0.01) and normotensive controls (*P* = 0.002; Figure 1D). No differences were seen for either sP- or sL-selectin concentrations (*P* > 0.05 for both; data not shown).

**Figure 2:**
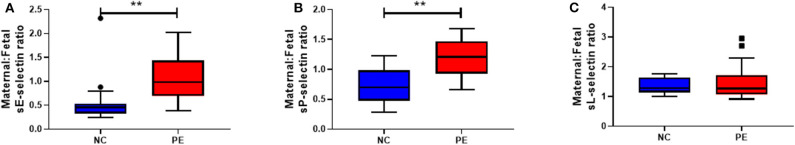
Maternal:fetal ratios of soluble (s) **(A)** E-selectin; **(B)** P-selection and **(C)** L-selectin concentrations between normotensive control (NC) and pre-eclamptic (PE) pregnancies. Data in all box plots are presented as median [interquartile ranges]; **P < 0.001.

Ratio between maternal and fetal sE-, sP- and sL-selectin was calculated (Figure 2). Significantly increased ratios were observed for both sE- (*P* = 0.001) and sP- (*P* = 0.02) selectin in pre-eclampsia, compared to normotensive controls. No differences were observed for sL-selectin (*P* > 0.05).

### Placental Selectin Protein Expression

**Figure 3:**
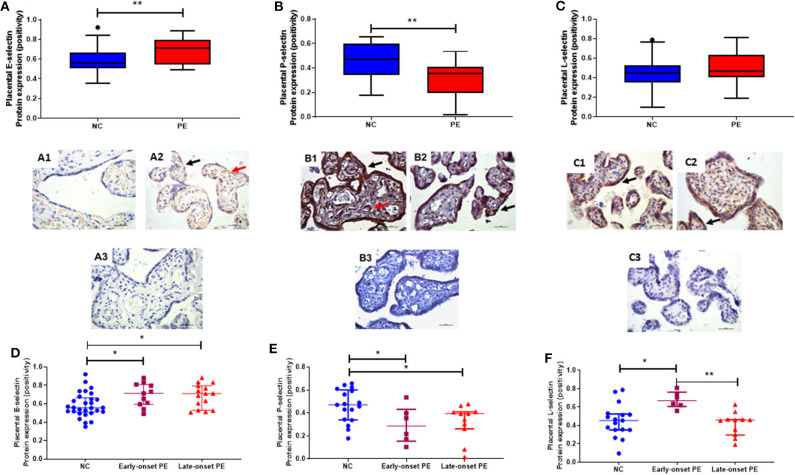
Localisation and quantification of placental **(A)** E-selectin; **(B)** P-selectin and **(C)** L-selectin in placenta from normotensive controls (NC (A1, B1, & C1); *n* = 17) and pre-eclampsia (PE (A2, B2, & C2); *n* = 17). Negative controls for each selectins are shown in A3, B3, & C3 respectively. In photomicrographs, positive cells appear in brown; magnification x400. Scale bars represent 100 μm. Protein expression was localized to the syncytiotrophoblast layer (black arrows) and some fetal vessels (red arrows). **(D)** E-selectin; **(E)** P-selectin and **(F)** L-selectin expression in placenta, when sub-grouped by NC, early-onset PE (diagnosis ≤ 34 weeks gestation; *n* = 6) and late-onset PE (>34 weeks gestation; *n* = 11). Data in all box plots are presented as median [interquartile ranges]; *P < 0.05; **P < 0.001.

Placental expression of all 3 selectins was determined and localization was mainly in the syncytiotrophoblast layer, stromal areas and some fetal vessel staining (Figure 3). Placental E-selectin expression was increased in pre-eclampsia (median [IQR], positivity; normotensive controls: 0.56 [0.51, 0.65]; pre-eclampsia: 0.71 [0.60, 0.80]; *P* = 0.008; Figure 3A), whereas P-selectin was reduced in pre-eclampsia (normotensive controls 0.47 [0.34, 0.59]; pre-eclampsia 0.35 [0.22, 0.41]; *P* = 0.008; Figure 3B). E-selectin expression levels were raised (*P* = 0.002), while P-selectin expression was reduced in both the early- and late-onset pre-eclampsia groups, compared to normotensive control samples (*P* < 0.05; Figures 3D,E).

Although no differences were observed between controls and pre-eclamptic women for L-selectin (*P* > 0.05; Figure 3C), when sub-grouped by onset of pre-eclampsia, a significant difference again became apparent the expression being increased in early-onset pre-eclampsia group expression compared to both normotensive controls (*P* = 0.008) and late-onset pre-eclampsia (*P* = 0.002; Figure 3F).

### Association of Placental Expression of Selectins With AT1R and AT2R

**Figure 4:**
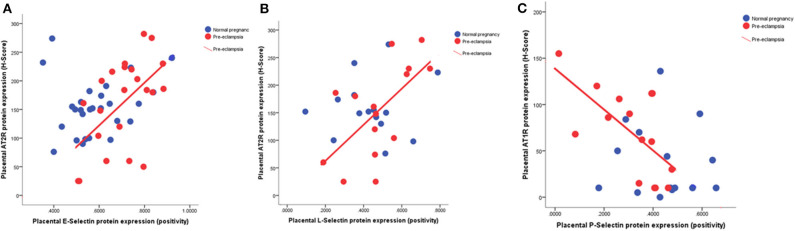
Scatter plots illustrating the positive relationship between **(A)** placental E-selectin (*r* = 0.55; *P* = 0.008) and **(B)** L-selectin (*r* = 0.61; *P* = 0.02) with angiotensin type 2 receptor (AT2R) and **(C)** the negative association between placental P-selectin (*r* = 0.68; *P* = 0.005) with angiotensin type 1 receptor (AT1R).

We compared placental expression of selectins with previously measured AT1R and AT2R placental protein expression. In the pre-eclampsia samples, but not those from normotensive women, placental expression of both E-selectin (*r* = 0.55; *P* = 0.008; Figure 4A) and L-selectin (*r* = 0.61; *P* = 0.02; Figure 4B) positively correlated with AT2R expression. In contrast, again only in the pre-eclampsia group, P-selectin displayed a negative association with AT1R expression (*r* = 0.68; *P* = 0.005; Figure 4C).

## Discussion

This is the first study to present data on all selectins in paired maternal/fetal plasma together with their placental expression in women with and without pre-eclampsia. It helps clarify the conflicting data found previously in relation to P- and L-selectin in pregnancy and pre-eclampsia.

The increased maternal sE-selectin in pre-eclampsia is in accordance with previous reports (22, 23, 25, 26). We have now shown that there is also increased placental E-selectin, reflecting the overall endothelial dysfunction associated with pre-eclampsia. Furthermore, the markedly increased concentrations of maternal sE-selectin, found only in the early-onset pre-eclampsia group (Figure 1D), presumably reflect the severity of the syndrome, as has been suggested by others (24). This is further supported by the finding that sE-selectin is increased prior to clinical diagnosis of pre-eclampsia (22, 38). Moreover, Chen et al., have shown that maternal sE-selectin was not associated with all cases of preterm birth, but specifically only when complicated by pre-eclampsia (39). The increased placental E-selectin expression we have shown corresponds with increased maternal sE-selectin and suggests that the placental dysfunction contributes to the endothelial damage in pre-eclampsia. Shaw et al., reported increased E-selectin mRNA expression in endothelial cell HUVECs and HEECs after incubation with maternal perfusates obtained from placenta of women with pre-eclampsia (40), which might amplify the underlying difference.

The interesting finding of reduced fetal sE-selectin in pre-eclampsia could either be a protective adaptation to limit the endothelial damage to the fetus, or reflects an underlying inability to synthesize E-selectin. Since E-selectin can be induced on fetal endothelial cells to the same extent as adult vessels by 32 weeks' gestation (17, 41) and the gestational age of the early-onset PE group was nearer 34 weeks, this supports the view that the difference is attributable to the pre-eclampsia pathology and not an immaturity of development. sE-selectin also contributes to homing in the endothelial progenitor cells and promotes tube formation in microvasculature as a repair mechanism (42). Given that there is reduced tubule formation in HUVECS isolated from pre-eclamptic pregnancies (43), this further suggests that the functional capacity of fetal endothelial cells is impaired in pre-eclampsia.

Nevertheless, the positive correlation with placental AT2R expression only in the pre-eclamptic samples supports the hypothesis of a protective mechanism; AT2R forms part of the “protective” arm of the RAS and is strongly upregulated following tissue damage such a vascular and neuronal injury (44). AT2R confers this role through its anti-inflammatory, anti-fibrotic and anti-apoptotic properties. Moreover, in lung tissues, which have many parallels with the placenta, Ang II treatment significantly increased E-selectin mRNA expression in vascular endothelial cells, further supporting the association (33).

We did not identify any differences in maternal sP-selectin between groups, which is in contrast to a previous study, which reported increased levels in pre-eclampsia (23, 26). A potential explanation for this difference lies in the later gestation age at sampling in our study when compared to the previous study. The lack of differences in sP-selectins between groups could have been due to our not measuring platelet sP-selectin levels. Nevertheless, by using whole plasma, we were able to measure all 3 selectins.

As with sE-selectins, the lower fetal P-selectin concentrations in the pre-eclamptic group could be a protective mechanism to limited endothelial damage to the fetus. The reduced placental P-selectin expression in pre-eclampsia could also contribute to lower fetal sP-selectin. In addition, the decreased P-selectin could also be explained by the known reduction in platelet numbers in pre-eclampsia (34). Furthermore, the inverse relationship with AT1R, suggests this is independent of AT1R expressed on platelets. Ang II can increase P-selectin expression within the vascular wall (45) and thus increase atherosclerotic plaque progression. Therefore, the reduced placental P-selectin expression in pre-eclampsia could be a feedback mechanism to limit this damage and is supported by this negative correlation with AT1R.

Inspection of Figures 2A,B led us to suggest that the ratio between maternal and fetal sE- or sP-selectin might differ between normotensive and pre-eclamptic women, and in early and late-onset pre-eclampsia. As Figure 3 shows, this is indeed the case, again emphasizing a possible protective mechanism.

The lack of differences observed between groups in sL-selectin in plasma may be because differences are only seen in specific cell types, for example the decreased levels in pre-eclampsia reported in neutrophils, T-cells, and monocytes (46). However, although not significant, there did appear to be a trend toward reduced maternal sL-selectin concentrations in the early-onset pre-eclamptic group. This is in line with previous studies where the majority of the pre-eclamptic samples measured were from early-onset pre-eclampsia (23, 47). To investigate this fully, future studies will examine whether any differences are observed when leukocytes and platelets are isolated and analyzed to confirm if the differences in this study are confirmed, or were masked by measuring in whole plasma. In contrast, there is increased placental expression in placenta from the early-onset pre-eclampsia group, which is not reflected in parallel increases in the maternal or fetal circulation. This again suggests a possible protective mechanism, further supported by the positive association seen with placental AT2R expression in the pre-eclamptic group. It has been suggested that these lower levels result in increased leukocyte activation as seen in some chronic inflammatory states (e.g., chronic artery disease (48)). We can thus speculate that the increased placental L-selectin in early-onset pre-eclampsia could lead to increased leucocyte activation and therefore atherosis in these placenta.

To conclude, this study is the first to report matched maternal, fetal and placental expression of all 3 selectins in pre-eclampsia. The increased E-selectins reflect the endothelial dysfunction, characteristic of pre-eclampsia. In contrast, the reduced P-selectins and the positive association with the placental AT2R with both E-and L-selectin in pre-eclampsia could be a protective mechanism to limit the endothelial dysfunction. Due to the roles of the selectins, this observational study further supports the importance of endothelial cells, platelets, and leucocytes in the pathogenesis of pre-eclampsia.

## Data Availability Statement

The datasets generated for this study are available on request to the corresponding author.

## Ethics Statement

The studies involving human participants were reviewed and approved by Nottingham University Hospital Ethics Committee. The patients/participants provided their written informed consent to participate in this study.

## Author Contributions

HM, LK, MO, and GE developed and performed measurement techniques, analyzed data, and wrote the manuscript. LK and HM collected samples and clinical information. FB directed the project, analyzed data, and assisted with writing the manuscript. All authors provided critical reviews of the manuscript.

## Conflict of Interest

The authors declare that the research was conducted in the absence of any commercial or financial relationships that could be construed as a potential conflict of interest.
